# Chaperonin GroEL/GroES Over-Expression Promotes Aminoglycoside Resistance and Reduces Drug Susceptibilities in *Escherichia coli* Following Exposure to Sublethal Aminoglycoside Doses

**DOI:** 10.3389/fmicb.2015.01572

**Published:** 2016-01-26

**Authors:** Lise Goltermann, Menachem V. Sarusie, Thomas Bentin

**Affiliations:** Department of Cellular and Molecular Medicine, University of CopenhagenCopenhagen, Denmark

**Keywords:** chaperonin, aminoglycosides, antibiotic resistance, protein misfolding, mRNA translation

## Abstract

Antibiotic resistance is an increasing challenge to modern healthcare. Aminoglycoside antibiotics cause translation corruption and protein misfolding and aggregation in *Escherichia coli*. We previously showed that chaperonin GroEL/GroES depletion and over-expression sensitize and promote short-term tolerance, respectively, to this drug class. Here, we show that chaperonin GroEL/GroES over-expression accelerates acquisition of streptomycin resistance and reduces susceptibility to several other antibiotics following sub-lethal streptomycin antibiotic exposure. Chaperonin buffering could provide a novel mechanism for emergence of antibiotic resistance.

## Introduction

Chaperones comprise an integral part of the protein folding machinery of bacterial cells and help maintain cellular homeostasis (Houry, 2001; Lin and Rye, 2006). Chaperones may also mask deleterious effects of mutations (Rutherford and Lindquist, 1998). Chaperonin GroEL/GroES promotes evolution of recombinant protein (Tokuriki and Tawfik, 2009) and a comparison of 446 bacterial genomes revealed that protein evolutionary rates in nature correlate positively with their dependency on GroEL/GroES (Bogumil and Dagan, 2010). Aminoglycoside (AG) antibiotics are known to promote translational misreading (Davies et al., 1964; Gorini and Kataja, 1964). Our lab (Goltermann et al., 2013) and others (Ling et al., 2012) have demonstrated that AG action promotes misfolding of newly synthesized protein. We have further demonstrated that GroEL/GroES over-expression countered nascent protein misfolding and promoted bacterial survival and growth in exponential cultures whereas chaperonin depletion sensitized cells to AG antibiotics (Goltermann et al., 2013). Consistent with chaperones playing key roles in the response to AG antibiotics, chaperone gene expression has been reported to be up-regulated in response to AG exposure across different bacterial species (Lin et al., 2005; Kohanski et al., 2008; Cardoso et al., 2010). Although not a mutagen in itself, the AG antibiotic streptomycin has been reported to induce mutations *via* translational misreading in streptomycin sensitive (*rpsL*^+^) wild type *E. coli* (Boe, 1992) and facilitate a mutator phenotype (Ren et al., 1999). Given that GroEL/GroES can promote short-term tolerance to AG antibiotics (Goltermann et al., 2013) and that streptomycin has indirect mutagenic properties (Boe, 1992; Ren et al., 1999), we speculated that chaperonin over-expression could buffer deleterious protein misfolding resulting from translational misreading during AG exposure and hence promote adaptation and resistance to AG antibiotics.

## Materials and methods

### Strains and plasmids

We used *E. coli* strain MG1655 (a gift from Stanley Brown, University of Copenhagen, strain #548 in our inventory) transformed with pACYC184 derived plasmids with protein expression driven by the arabinose inducible *Pbad* promoter. The following plasmids were used: pGroEL/GroES (p544 in our inventory) expressing GroEL/GroES, pΔGroEL/GroES (p730-c1 in our inventory) deleted for the *groS-groL* genes (Goltermann et al., 2013), and pGFP (p488-c1 in our inventory) expressing GFPuv (Crameri et al., 1996) containing the F64L, S65T (Heim et al., 1995; Cormack et al., 1996) mutations (Goltermann et al., 2013). pGroEL/GroES is commercially available from Takara Biosciences as pGro7 (Nishihara et al., 1998). The p730-c1 and p488-c1 plasmids were previously described (Goltermann et al., 2013). The construction of p730-c1 was erroneously described and we therefore describe this plasmid in detail: Plasmid p730-c1 (ΔGroEL/GroES) was generated by inverse PCR using pGroEL/GroES as a template and 5′-phosphorylated primers otb674 (5′P-TTGAGAAAGTCCGTATCTGTTATGGGTG) and otb695 (5′P-GCCCTGCACCTCGCAGAAATAAAC). The PCR product/template mix was *Dpn*I treated to remove template. The PCR amplicon was circularized with DNA ligase and transformed into *E. coli*. The resulting construct was characterized by restriction mapping using *EcoR*I and *Hind*III yielding bands compatible with the expected fragment sizes (1947 and 1523 bp) and by sequencing across the *groS-groL* deletion using otb710 (CAAAAGCGTACAGTTCAGGCG).

### Susceptibility assay and MIC value determinations

Over-night MG1655/plasmid cultures in LB containing 40 μg/ml chloramphenicol (LBC; for plasmid maintenance) and 0.02% L-arabinose (for GroEL/GroES induction) were diluted into fresh LBC containing arabinose and sub-inhibitory concentrations of selection antibiotic (12–14 μg/ml streptomycin, 15 μg/ml spectinomycin, or 18 μg/ml ampicillin (see Figure S1) followed by continued growth over-night. Cultures were diluted to OD595 nm = 0.003 in fresh medium, and the resulting cultures were grown in Erlenmeyer flasks at 37°C, 180 rpm ON. The cultures were passaged every 24 h over the course of 3 days. At each passage, aliquots of out-grown cultures were removed normalized to 1 OD_595 nm_ per ml and 100 μl of undiluted culture as well as ten-fold dilutions were plated on LBC-agar containing inhibitory amounts of selection antibiotic (40 μg/ml streptomycin, 80 μg/ml spectinomycin, or 60 μg/ml ampicillin) to determine the number of resistant colony forming units (cfu), and on LBC-agar to determine the total cfu count. Drug tolerant cfu and total cfu were thus determined on plates without L-arabinose. Growth inhibitory concentrations of selection antibiotic were determined as the concentration that did not allow visible growth following 24 h incubation at 37°C of an unexposed culture. Following over-night incubation, cfu were counted manually. Examples of colonies growing on inhibitory selection antibiotic (streptomycin) were re-streaked on fresh plates to determine whether the reduced drug susceptibility was stably inherited. Drug susceptibility to streptomycin and other antibiotics was investigated further by growth in LBC liquid culture using microtiter plates and using the indicated antibiotic concentrations (Table 1). MIC determinations were done by replica plating from a fresh over-night culture using a 48-pin tool (frogger) into 200 μl of LBC and the desired test antibiotic (Table 2). Plates were double taped to avoid evaporation and scored for ±growth after over-night incubation at 37°C at 900 rpm in a Heidolph microplate incubator. Sequencing of the chromosomal *rpsL* gene in streptomycin resistant isolates was done using primer otb722 following PCR amplification of the *rpsL* locus using forward primer otb721 (TCTGCGTAATGCCCCCATTAAGG) and reverse primer otb722 (AACTTCGGATCCGGCAGAATTTTAC).

**Table 1:** **Susceptibility to other antibiotics of streptomycin selected isolates**.

	**pGroEL/GroES**	**pΔGroEL/GroES**
Streptomycin	100% (95/95)	100% (127/127)
Ampicillin	15% (14/95)	2% (3/127)
Tetracycline	0% (0/95)	0% (0/127)
Spectinomycin	19% (18/95)	3% (4/127)
Kanamycin	9% (9/95)	1% (1/127)
Amp/Spc/Str	5% (5/95)	0 (0/127)
Spc/Kan/Str	4% (4/95)	0 (0/127)

*Sensitivity to other antibiotics of isolates growing on inhibitory streptomycin plates from pGroEL/GroES and pΔGroEL/GroES transformed MG1655 cultures after 24 h of sub-inhibitory streptomycin selection. Streptomycin, Str (100 μg/ml), Ampicillin, Amp (100 μg/ml), Tetracycline (10 μg/ml), Spectinomycin, Spc (50 μg/ml, Spc), Kanamycin, Kan (50 μg/ml, Kan)*.

**Table 2:** **MIC values of streptomycin selected pGroEL/GroES isolates**.

	**Control**	**Isolates**
Streptomycin (μg/ml)	4	≥128
Kanamycin (μg/ml)	8	128
Spectinomycin (μg/ml)	16	64
Ampicillin (μg/ml)	16	64
Tetracycline (μg/ml)	2	2

*“Isolates” and “Control” designate pGroEL/GroES MG1655 cultures that had or had not been exposed to streptomycin selection prior to MIC determination, respectively. Numbers are based on the experiment shown in Table S2*.

## Results and discussion

In order to determine whether GroEL/GroES over-expression promotes resistance to AG antibiotics, we examined drug susceptibilities of MG1655 over-expressing the chaperonin complex following exposure to sub-lethal concentrations of various “selection antibiotics”: (i) Streptomycin, an aminoglycoside causing ribosomal misreading and protein misfolding, (ii) spectinomycin, a bacteriostatic antibiotic that causes translational blockage without misreading or protein misfolding, and (iii) ampicillin, which targets the peptidoglycan layer and therefore is not expected to impact protein folding. The antibiotic concentrations used were based on a titration, which showed similar growth phenotypes among the differently used antibiotics (Figure S1). We used the *E. coli* strain MG1655 transformed with one of three arabinose inducible plasmids. One transformant contained the plasmid pGroEL/GroES (Nishihara et al., 1998) expressing chaperonin GroEL/GroES. As controls, we used MG1655 transformed with pΔGroEL/GroES in which the entire *groS-groL* coding region was deleted (empty control) and MG1655 transformed with pGFP, a plasmid that expresses GFP (over-expression control). Cultures were passaged with arabinose inducer for 3 days in total (Figure S2) and drug susceptibility was then determined without chaperonin induction.

**Figure 1:**
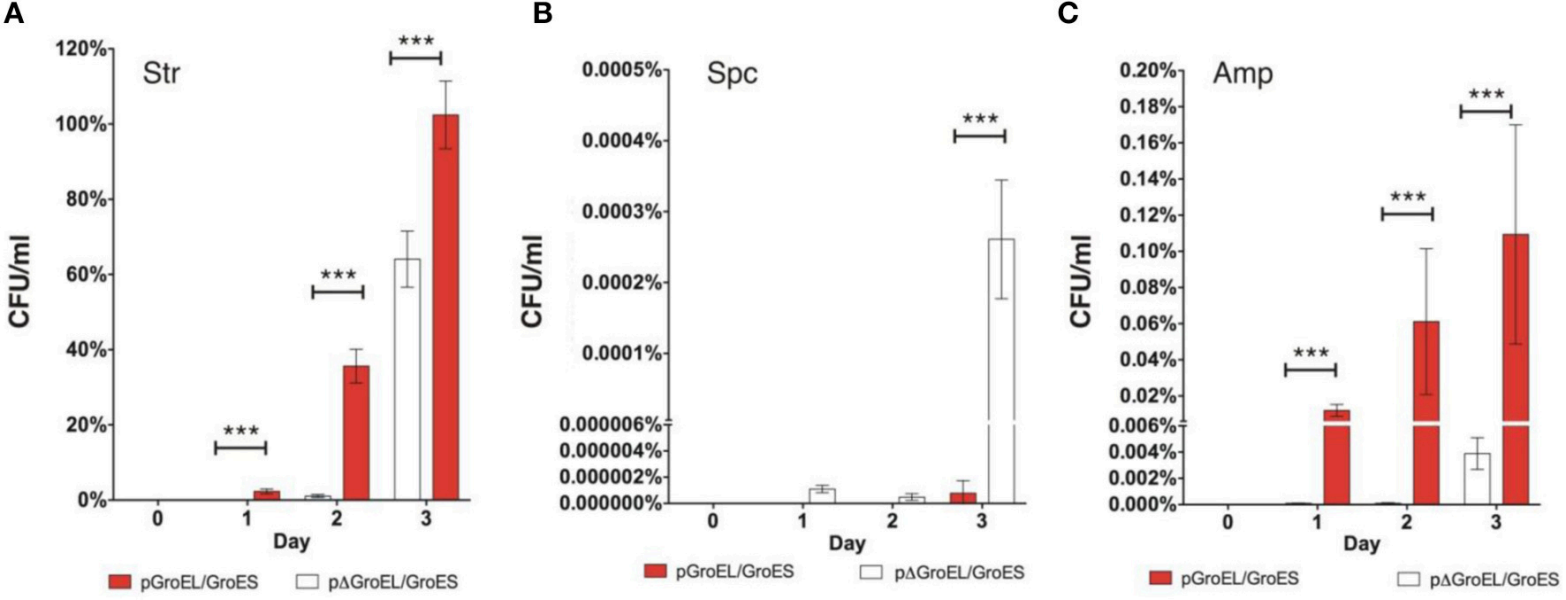
**Chaperonin dependent antibiotic resistance development following sub-inhibitory selection**. Percentage of colonies growing on inhibitory concentrations of the indicated antibiotics used for selection with or without over-expression of GroEL/GroES (pGroEL/GroES), or an empty control plasmid (pΔGroEL/GroES). The number of cfu growing on inhibitory antibiotic plates was normalized to total cfu count (Each data point consists of 2-4 experimental repeats each composed of 2-4 technical repeats. Error bars show SD). Day 1: pGroEL/GroES vs. pΔGroEL/GroES *p* < 0.0001 (^***^), day 2: pGroEL/GroES vs. pΔGroEL/GroES *p* < 0.0001 (^***^), day 3: pGroEL/GroES vs. pΔGroEL/GroES *p* < 0.0001 (^***^). Note the split y-axis used to enable visualization of large differences within the same graph. **(A)** Str, streptomycin; **(B)** Spc, spectinomycin; **(C)** Amp, ampicillin.

The pGroEL/GroES transformed strain showed a rapid reduction of drug susceptibility following streptomycin selection as determined by the percentage of cfu growing on plates containing an inhibitory streptomycin concentration (Figure 1A). In fact, the pGroEL/GroES transformed culture was virtually purged for streptomycin sensitive bacteria in only 3 days. MG1655 transformed with pΔGroEL/GroES also showed reduced drug susceptibility following growth in sub-lethal streptomycin but this was much less pronounced requiring extended exposure (Figure 1A). This tendency also holds true in absolute numbers (Figure S3B), even though the pGroEL/GroES transformed strain grew to a lower cell density on day 1 of selection (see legend to Figure S2). Similar to the pΔGroEL/GroES transformed strain, streptomycin tolerance in MG1655/pGFP also developed slowly (Figure S3), indicating that protein over-expression in itself did not enhance antibiotic adaptation. We conclude that acceleration of adaptation toward streptomycin was a result of chaperonin action.

Colonies derived following sub-lethal streptomycin selection and growth on inhibitory streptomycin plates, were incubated in liquid culture containing 100 μg/ml streptomycin and all showed growth (Table 1). This suggests that these colonies were not only temporarily tolerant but heritably resistant toward streptomycin.

Among the 95 chaperonin over-expressing isolates, 17 randomly selected colonies from the first day of selection were sequenced for changes in the *rpsL* gene encoding ribosomal protein S12. Mutations in the S12 protein that confer resistance to streptomycin are known. Two independent point mutations giving rise to an arginine to serine mutation at codon 85 (R85S) and a lysine to arginine mutation at codon 87 (K87R) were found (Table S1). A streptomycin sensitive control, which had not been exposed to streptomycin selection, was sequenced and revealed the wild type *rpsL* gene sequence (Table S1). The K87R mutation confers resistance to streptomycin (Timms et al., 1992). We have not found a prior description of the specific R85S mutation. These observations show that GroEL/GroES over-expression can enable accelerated resistance acquisition during sub-lethal streptomycin selection and that this resistance is genetically based.

A number of resistant isolates identified on day 1 after streptomycin or ampicillin selection were tested for susceptibility to other antibiotics at or above the MIC value of the parent strain (Table 1). Interestingly, several pGroEL/GroES isolates resistant to streptomycin were found to show reduced susceptibility to one (ampicillin, spectinomycin, or kanamycin) or two (spectinomycin and kanamycin or spectinomycin and ampicillin) additional antibiotics. Susceptibility to tetracycline was unaltered.

To explore drug susceptibilities further, we repeated the selection experiment in sub-lethal streptomycin concentrations and picked fresh pGroEL/GroES isolates growing on inhibitory streptomycin plates. These isolates were grown ON in a 96 well format. We then determined MIC values by replica plating and growth ON in media (Table 2; Table S2). Compared to the control (included in duplicate), which had not been subjected to selection, isolates were observed to show reduced drug susceptibilities to streptomycin (as expected), to kanamycin, and to a lesser extent to spectinomycin and ampicillin. As above, tetracycline sensitivity was unaltered. MIC values are known to be sensitive to the inoculum density and since these isolates were inoculated using a pin tool (Table S2) whereas the data presented in Table 1 was obtained following inoculation of cfu, we did not expect identical numbers. Nevertheless, we observe a clear trend that isolates over-expressing GroEL/GroES during growth with sub-lethal streptomycin exposure rapidly acquired reduced susceptibility to the selection drug and also to additional drugs.

Selection with spectinomycin resulted in a very low fraction of colonies showing reduced susceptibility to the drug even after 3 days of selection, regardless of chaperonin status (Figure 1B). Cultures grown without antibiotic selection did not yield any colonies under any of the tested conditions indicating that spontaneous formation of resistant isolates w/o antibiotic selection was under our detection limit (estimated to be ~1 cfu in 10^8^ cells).

Selection with ampicillin produced only few colonies showing reduced ampicillin susceptibility (Figure 1C), and only 1–4% of these isolates showed reduced susceptibility to an additional antibiotic (Table S3). Unexpectedly, the pGroEL/GroES transformed strain showed a small but significant reduction of susceptibility to ampicillin as compared to MG1655 harboring pΔGroEL/GroES (Figure 1C) even though ampicillin action targets the peptidoglycan layer and does not involve protein misfolding. We do not know the mechanism(s) connecting chaperonin over-expression to reduced ampicillin susceptibility. One speculative mechanism could involve GroEL/GroES dependent accelerated protein evolution, as described for GroES/GroEL substrates *in vitro* (Tokuriki and Tawfik, 2009) and *in vivo* (Bogumil and Dagan, 2010). To this end, GroEL/GroES is a known cytosolic chaperone, but it nevertheless does interact with a few membrane proteins (Kerner et al., 2005) and could chaperone a wider collection of proteins than those identified by interaction analyses (Chapman et al., 2006).

We repeated the selection experiment using two other AG antibiotics: Kanamycin and gentamicin (Figure S4). Resistance toward these antibiotics occurs infrequently as compared with streptomycin. Nevertheless, like in the case of streptomycin, GroEL/GroES over-expressing bacteria grown under sub-inhibitory kanamycin (14 μg/ml) or gentamicin (1.5 μg/ml) concentrations showed more colonies on inhibitory plates as compared with the ΔGroEL/GroES strain following plating on plates containing 60 μg/ml kanamycin or 10 μg/ml gentamicin, respectively. Compared to the streptomycin selection experiment, colonies from kanamycin or gentamicin selection were smaller indicating slower growth. The observation that more cfu grew following selection with GroEL/GroES over-expression, however, suggests that chaperonin over-expression also promotes reduced drug susceptibilities to these members of the AG class of antibiotics.

The present results reveal that chaperonin action can accelerate resistance development to streptomycin following exposure to sub-lethal streptomycin concentrations and simultaneously reduce drug susceptibilities to other antibiotics to which the bacteria had not previously been exposed. These observations are compatible with chaperonin over-expression conferring an increased fitness due to reduced protein misfolding during exposure to AG. In turn, this enables selection for reduced drug susceptibility, which is accelerated due to the mutator phenotype resulting from streptomycin exposure (Ren et al., 1999). Such selected strains would also have acquired numerous additional mutations. Mutations reducing influx or increasing efflux (Pagès et al., 2008; Nikaido and Pagès, 2012) could explain reduced susceptibility to multiple antibiotics.

Together, the results suggest that chaperonins could enable formation of complex antibiotic susceptibility phenotypes from a single AG exposure regime. We propose a model, where GroEL/GroES over-expression reduce protein misfolding, increase fitness, expand the “mutational space,” and provide a window of opportunity for bacteria to acquire resistance or tolerance and hence evade drug mediated killing.

## Author contributions

LG and TB conceived the project. LG, MVS, and TB designed experiments. MVS and LG performed experiments. MVS, LG, and TB analyzed the data. LG and TB and wrote the manuscript.

### Conflict of interest statement

The authors declare that the research was conducted in the absence of any commercial or financial relationships that could be construed as a potential conflict of interest.
