# Supplementary material for: Chaperonin GroEL/GroES Over-Expression Promotes Aminoglycoside Resistance and Reduces Drug Susceptibilities in Escherichia coli Following Exposure to Sublethal Aminoglycoside Doses
Source: Front Microbiol. 2016 Jan 26;6:1572. doi: 10.3389/fmicb.2015.01572 (PMC4726795; doi:10.3389/fmicb.2015.01572)
Supplement: Supplementary file 1 [file Table1.pdf]

1.2 Supplementary Tables

Description	Alignment	Codon change
rpsL ecocyc.org	GGTCGTGTTAAAGAC<br>| | | | | G R V K D	
Clone 1 (control)	GGTCGTGTTAAAGAC	No changes
Clone 2	GGTAGTGTTAAAGAC	R85S
Clone 3	GGTCGTGTTAGAGAC	K87R
Clone 4	GGTCGTGTTAGAGAC	K87R
Clone 5	GGTCGTGTTAGAGAC	K87R
Clone 6	GGTCGTGTTAGAGAC	K87R
Clone 7	GGTCGTGTTAGAGAC	K87R
Clone 8	GGTCGTGTTAGAGAC	K87R
Clone 9	GGTCGTGTTAGAGAC	K87R
Clone 10	GGTCGTGTTAGAGAC	K87R
Clone 11	GGTCGTGTTAGAGAC	K87R
Clone 12	GGTCGTGTTAGAGAC	K87R
Clone 13	GGTCGTGTTAGAGAC	K87R
Clone 14	GGTCGTGTTAGAGAC	K87R
Clone 15	GGTCGTGTTAGAGAC	K87R
Clone 16	GGTCGTGTTAGAGAC	K87R
Clone 17	GGTCGTGTTAGAGAC	K87R
Clone 18	GGTCGTGTTAGAGAC	K87R

Table S1. Sequencing of streptomycin resistant clones at the *rpsL* locus. 17 GroEL/GroES overexpressing clones isolated as growing on 40 µg/ml streptomycin following sublethal streptomycin selection growth were sequenced to identify possible changes in the *rpsL* gene. All contained single nucleotide substitutions leading to a codon change compared to the parent strain. The region encoding amino acids 84-88 of protein S12 are shown.
