# Supplementary material for: Chaperonin GroEL/GroES Over-Expression Promotes Aminoglycoside Resistance and Reduces Drug Susceptibilities in Escherichia coli Following Exposure to Sublethal Aminoglycoside Doses
Source: Front Microbiol. 2016 Jan 26;6:1572. doi: 10.3389/fmicb.2015.01572 (PMC4726795; doi:10.3389/fmicb.2015.01572)
Supplement: Supplementary file 2 [file Table2.pdf]

	Str (rep 1)		Str (rep 2)		Kan (rep 1)		Kan (rep 2)	
Antibiotic (ug/ml)	controls	isolates	controls	isolates	controls	isolates	controls	isolates
0	2 (2)	94 (94)	2 (2)	94 (94)	2 (2)	94 (94)	2 (2)	94 (94)
1	2 (2)	94 (94)	2 (2)	94 (94)	2 (2)	94 (94)	2 (2)	94 (94)
2	2 (2)	94 (94)	2 (2)	94 (94)	2 (2)	94 (94)	2 (2)	94 (94)
4	0 (2)	94 (94)	0 (2)	94 (94)	2 (2)	94 (94)	2 (2)	94 (94)
8	0 (2)	94 (94)	0 (2)	94 (94)	0 (2)	94 (94)	0 (2)	94 (94)
16	0 (2)	94 (94)	0 (2)	94 (94)	0 (2)	92 (94)	0 (2)	91 (94)
32	0 (2)	94 (94)	0 (2)	91 (94)	0 (2)	55 (94)	0 (2)	49 (94)
64	0 (2)	94 (94)	0 (2)	91 (94)	0 (2)	1 (94)	0 (2)	1 (94)
128	0 (2)	1 (94)	0 (2)	0 (94)	0 (2)	0 (94)	0 (2)	0 (94)

	Spc (rep 1)		Spc (rep 2)		Amp (rep 1)		Amp (rep 2)		Tet (rep 1)	
Antibiotic (ug/ml)	controls	isolates	controls	isolates	controls	isolates	controls	isolates	controls	isolates
0	2 (2)	94 (94)	2 (2)	94 (94)	2 (2)	94 (94)	2 (2)	94 (94)	2 (2)	94 (94)
1	2 (2)	94 (94)	2 (2)	94 (94)	2 (2)	94 (94)	2 (2)	94 (94)	2 (2)	94 (94)
2	2 (2)	94 (94)	2 (2)	94 (94)	2 (2)	94 (94)	2 (2)	94 (94)	0 (2)	0 (94)
4	2 (2)	94 (94)	2 (2)	94 (94)	2 (2)	94 (94)	2 (2)	94 (94)	0 (2)	0 (94)
8	2 (2)	94 (94)	2 (2)	94 (94)	2 (2)	94 (94)	2 (2)	94 (94)	0 (2)	0 (94)
16	0 (2)	88 (94)	0 (2)	93 (94)	0 (2)	89 (94)	0 (2)	93 (94)	0 (2)	0 (94)
32	0 (2)	17 (94)	0 (2)	82 (94)	0 (2)	1 (94)	0 (2)	23 (94)	0 (2)	0 (94)
64	0 (2)	0 (94)	0 (2)	0 (94)	0 (2)	0 (94)	0 (2)	0 (94)	ND	ND
128	0 (2)	0 (94)	0 (2)	0 (94)	0 (2)	0 (94)	0 (2)	0 (94)	ND	ND

Table S2. MIC determination of streptomycin selected isolates against the indicated antibiotics. Isolates: MG1655/pGroEL/GroES cultures subjected to sub-lethal selection (12 ug/ml streptomycin) growth with GroEL/GroES induction prior to MIC determination. Controls: MG1655/pGroEL/GroES cultures that were not subjected to selection growth in streptomycin prior to MIC determination. MIC determinations were done without recombinant chaperonin induction. Nomenclature: numbers w/o brackets indicate growing cultures; numbers in bracket indicates total number of cultures examined.
