# Supplementary material for: Chaperonin GroEL/GroES Over-Expression Promotes Aminoglycoside Resistance and Reduces Drug Susceptibilities in Escherichia coli Following Exposure to Sublethal Aminoglycoside Doses
Source: Front Microbiol. 2016 Jan 26;6:1572. doi: 10.3389/fmicb.2015.01572 (PMC4726795; doi:10.3389/fmicb.2015.01572)
Supplement: Supplementary file 3 [file Table3.pdf]

	pGroEL/GroES	pΔGroEL/GroES
Ampicillin	100% (95/95)	100% (95/95)
Streptomycin	1% (1/95)	0% (0/95)
Tetracycline	0% (0/95)	0% (0/95)
Spectinomycin	4% (4/95)	0% (0/95)
Kanamycin	0% (0/95)	0% (0/95)

Table S3. Susceptibility to additional antibiotics of isolates derived following ampicillin selection. Colonies isolated as growing on plates containing inhibitory ampicillin concentration after 24 h of sub-inhibitory antibiotic selection in 18 µg/ml ampicillin were tested on plates containing 100 µg/ml ampicillin, 100 µg/ml streptomycin, 10 µg/mltetracycline, 50 µg/ml spectinomycin, or 50 µg/ml kanamycin.
