# Supplementary material for: Chaperonin GroEL/GroES Over-Expression Promotes Aminoglycoside Resistance and Reduces Drug Susceptibilities in Escherichia coli Following Exposure to Sublethal Aminoglycoside Doses
Source: Front Microbiol. 2016 Jan 26;6:1572. doi: 10.3389/fmicb.2015.01572 (PMC4726795; doi:10.3389/fmicb.2015.01572)
Supplement: Supplementary file 4 [file Image1.pdf]

Supplementary Material

Article Title

Lise Goltermann, Viktor Menachem Sarusie, and Thomas Bentin*

*** Correspondence:** Thomas Bentin: bentin@sund.ku.dk

1.1 Supplementary Figures

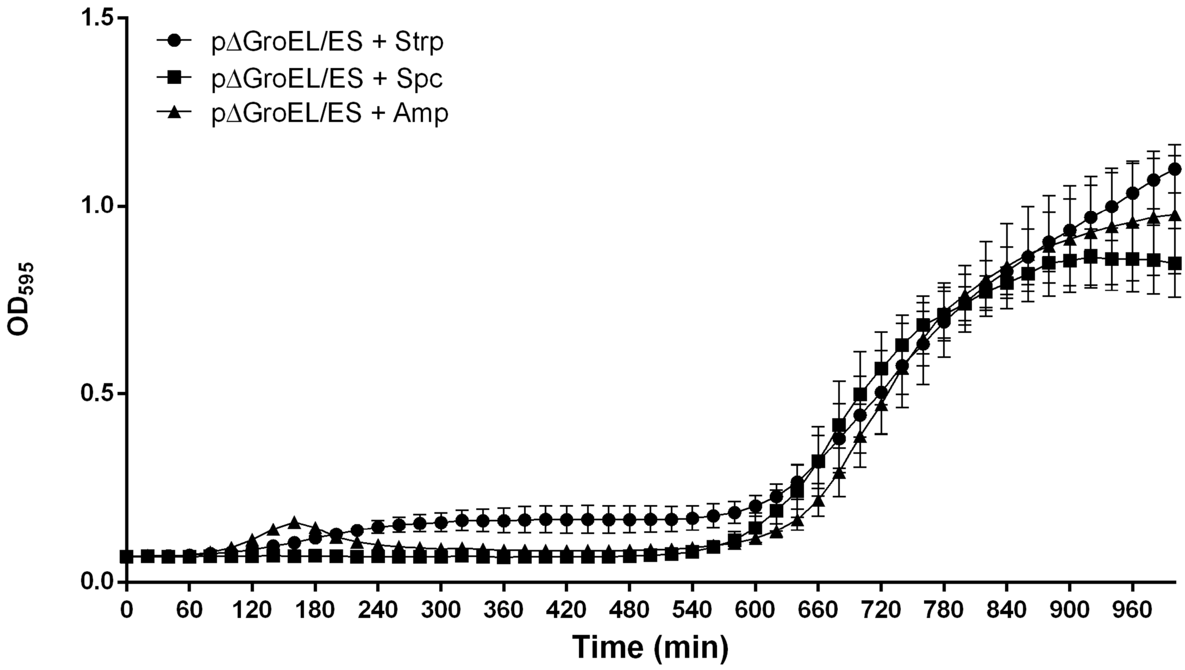

Figure S1. Growth curves for the indicated MG1655 transformants grown with sub-inhibitory antibiotic concentrations (14 µg/ml streptomycin (Str), 18 µg/ml ampicillin (Amp) or 15 µg/ml spectinomycin (Spc)) chosen to obtain similar growth (n = 5).
