# Supplementary material for: Chaperonin GroEL/GroES Over-Expression Promotes Aminoglycoside Resistance and Reduces Drug Susceptibilities in Escherichia coli Following Exposure to Sublethal Aminoglycoside Doses
Source: Front Microbiol. 2016 Jan 26;6:1572. doi: 10.3389/fmicb.2015.01572 (PMC4726795; doi:10.3389/fmicb.2015.01572)
Supplement: Supplementary file 5 [file Image2.pdf]

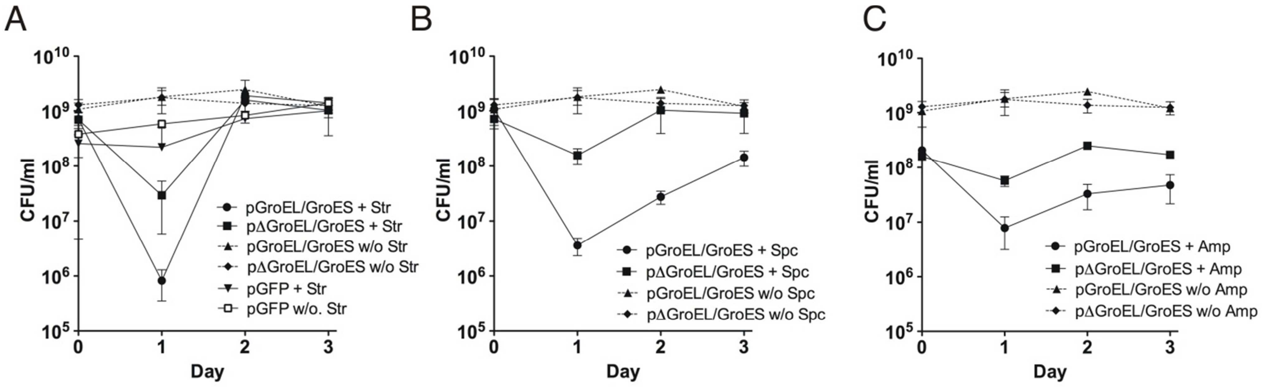

Figure S2. Cell density in over-night cultures following antibiotic selection. A) 14 µg/ml streptomycin (Str), **B)** 15 µg/ml spectinomycin (Spc), **C)** 18 µg/ml ampicillin (Amp). Optical density was measured at OD595 nm. We note a significant CFU/ml drop on the first day of selection for the strain harboring pGroEL/GroES and to a lesser extent for the pΔGroEL/GroES deletion control. In previous short term experiments using exponential cultures, we did not observe such a drop (Goltermann et al., 2013). We cannot presently explain this discrepancy but note that the physiological state of cells starting from a dilute inoculum derived from a stationary phase over-night culture (this work) and exponential cells (Goltermann et al., 2013) differ substantially and that “tying up” growth-essential translation components through recombinant protein expression may impact cells differently in such circumstances.
