# Supplementary material for: Chaperonin GroEL/GroES Over-Expression Promotes Aminoglycoside Resistance and Reduces Drug Susceptibilities in Escherichia coli Following Exposure to Sublethal Aminoglycoside Doses
Source: Front Microbiol. 2016 Jan 26;6:1572. doi: 10.3389/fmicb.2015.01572 (PMC4726795; doi:10.3389/fmicb.2015.01572)
Supplement: Supplementary file 6 [file Image3.pdf]

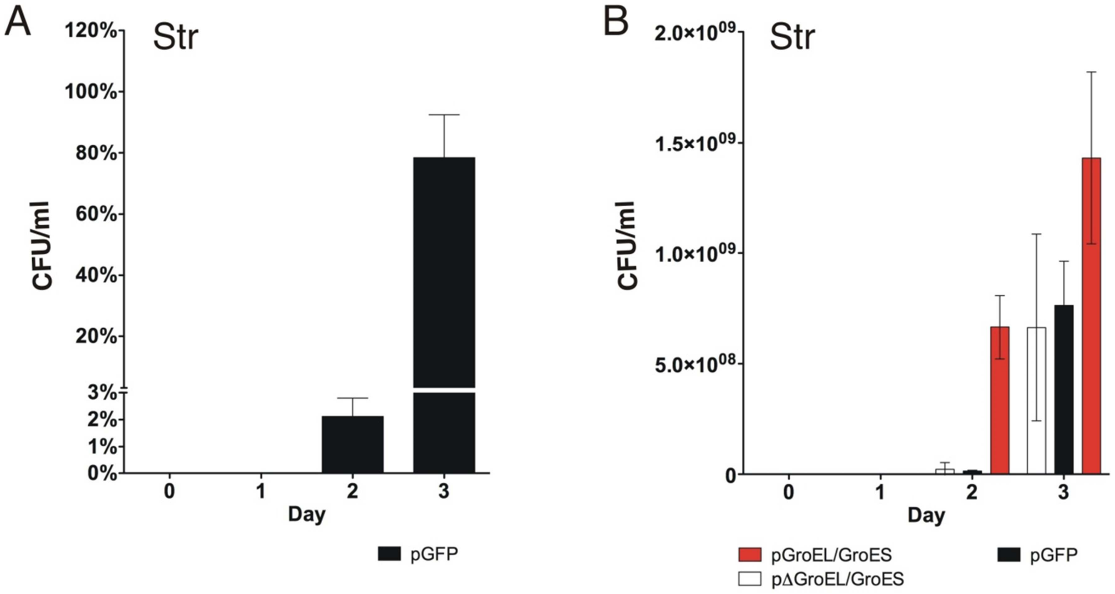

Figure S3. **Antibiotic susceptibilities following sub-inhibitory streptomycin selection.** A) Percentage of GFP overexpressing colonies growing on inhibitory streptomycin plates (Str) after exposure to sub-lethal streptomycin growth. The number of cfu growing on LBC-agar containing inhibitory streptomycin was normalized to total cfu count on plates without streptomycin ($n \geq 4$). B) Absolute number of colonies growing on inhibitory (40 μg/ml) streptomycin following selection. In A and B cells were grown with or without overexpression of GroEL/GroES (pGroEL/GroES), an empty control plasmid (pΔGroEL/GroES), or GFP (pGFP).
