# Supplementary material for: Chaperonin GroEL/GroES Over-Expression Promotes Aminoglycoside Resistance and Reduces Drug Susceptibilities in Escherichia coli Following Exposure to Sublethal Aminoglycoside Doses
Source: Front Microbiol. 2016 Jan 26;6:1572. doi: 10.3389/fmicb.2015.01572 (PMC4726795; doi:10.3389/fmicb.2015.01572)
Supplement: Supplementary file 7 [file Image4.pdf]

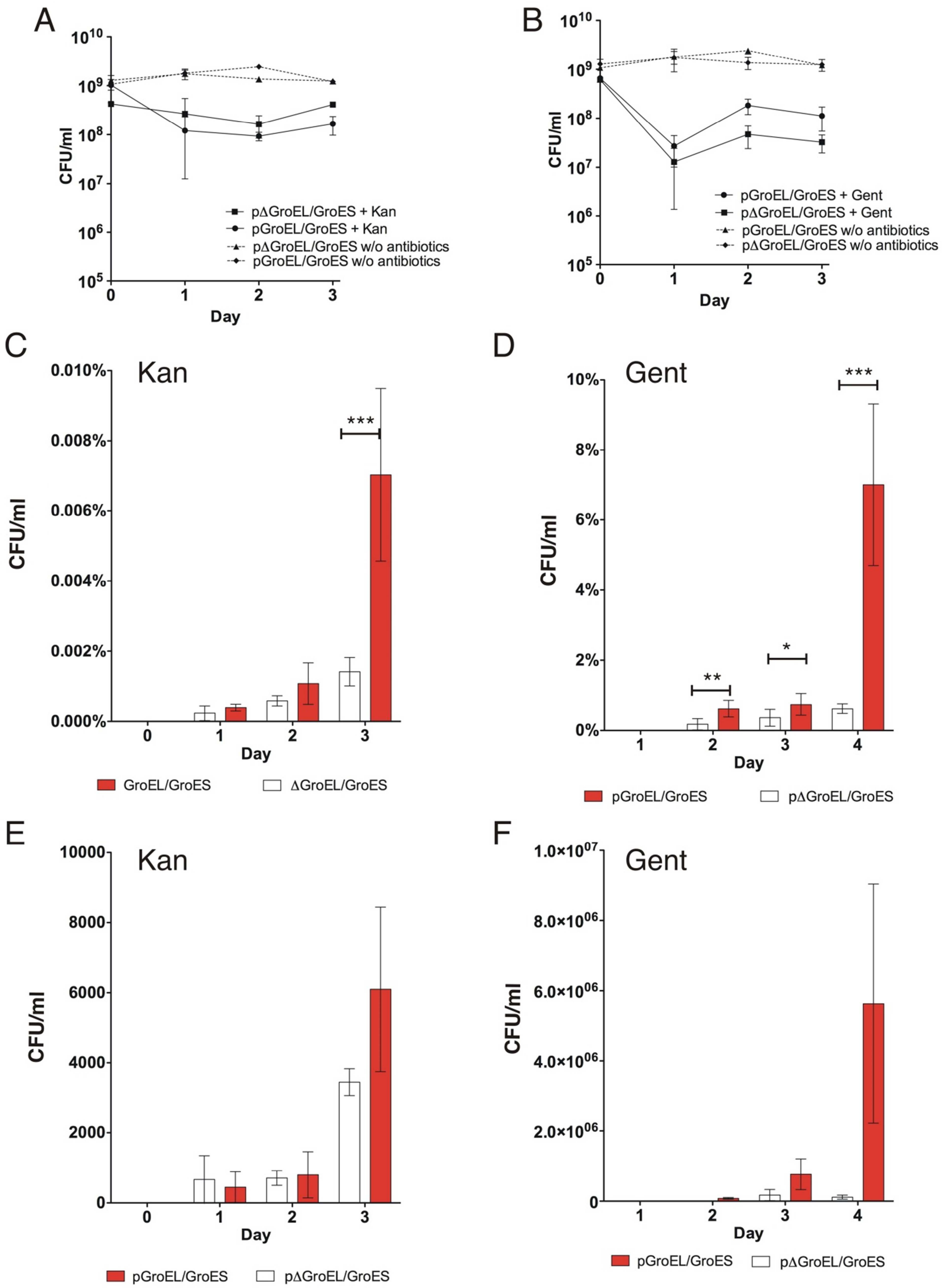
A
B
C
D
E
F
CFU/ml
Day
pΔGroEL/GroES + Kan
pGroEL/GroES + Kan
pΔGroEL/GroES w/o antibiotics
pGroEL/GroES w/o antibiotics
pGroEL/GroES + Gent
pΔGroEL/GroES + Gent
pGroEL/GroES w/o antibiotics
pΔGroEL/GroES w/o antibiotics
Kan
Gent
GroEL/GroES
ΔGroEL/GroES
pGroEL/GroES
pΔGroEL/GroES

Figure S4. Cell density and chaperonin dependent antibiotic susceptibility following sub-inhibitory aminoglycoside selection. A-B) Optical density of the indicated cultures following over-night incubation in LBC with sub-lethal antibiotic selection. **A)** 14 µg/ml kanamycin (Kan), **B)** 1.5 µg/ml gentamicin (Gent). Optical density was measured at OD595 nm. **C-D)** Percentage of colonies growing on 60 µg/ml kanamycin (C) or 10 µg/ml gentamicin (D) following growth with sub-lethal antibiotic selection with or without overexpression of GroEL/GroES (pGroEL/GroES), or an empty control plasmid (pΔGroEL/GroES). The number of colonies growing on plates with inhibitory AG as indicated was normalized to total cfu count derived from plates without AG antibiotic ($n = 2$). Day 1, Kan: pGroEL/GroES vs. pΔGroEL/GroES $p<0.0001$ (***); Day 1, Gent: pGroEL/GroES vs. pΔGroEL/GroES $p<0.0030$ (**), day 2, Gent: pGroEL/GroES vs. pΔGroEL/GroES $p<0.0246$ (*), day 3, Gent: pGroEL/GroES vs. pΔGroEL/GroES $p<0.0001$ (***). **E-F)** Data from C and D in absolute numbers.
